# Molecular mechanisms mediated by liquid-liquid phase separation in chronic liver disease progression

**DOI:** 10.1016/j.isci.2025.113843

**Published:** 2025-10-24

**Authors:** Xinran Qiu, Haoyuan Tian, Yuanyuan Gao, Junrui Wang, Zhengyang Bao, Ningyu Qiu, Feng Zhang, Zili Zhang, Feixia Wang, Shizhong Zheng, Jiangjuan Shao

**Affiliations:** 1 Jiangsu Key Laboratory for Pharmacology and Safety Research of Chinese Materia Media, Nanjing University of Chinese Medicine, Nanjing 210023, China

**Keywords:** Biological sciences, Molecular biology, Cell biology

## Abstract

Liquid-liquid phase separation (LLPS) is a cellular process driven by multivalent interactions, forming dynamic biomolecular condensates containing proteins, RNAs, and other molecules. LLPS plays a pivotal role in processes such as signal transduction, gene expression, autophagy, and cellular stress responses. The dysregulation of LLPS is linked to chronic liver diseases (CLDs), particularly non-alcoholic fatty liver disease (NAFLD), liver fibrosis, and hepatocellular carcinoma (HCC). LLPS profoundly mediates the pathological evolution of these diseases by regulating key mechanisms, including lipid metabolism, inflammatory responses, and cell death. This review highlights the central role of LLPS in NAFLD progression, liver fibrosis, and HCC transformation. Furthermore, it evaluates the feasibility of targeting LLPS as a therapeutic strategy, proposing innovative approaches such as small-molecule inhibitors, protein modification regulators, and RNA interference to restore LLPS homeostasis. These strategies hold the potential to mitigate disease progression and prevent the transition to fibrosis and liver cancer.

## Graphical abstract

**Figure:**
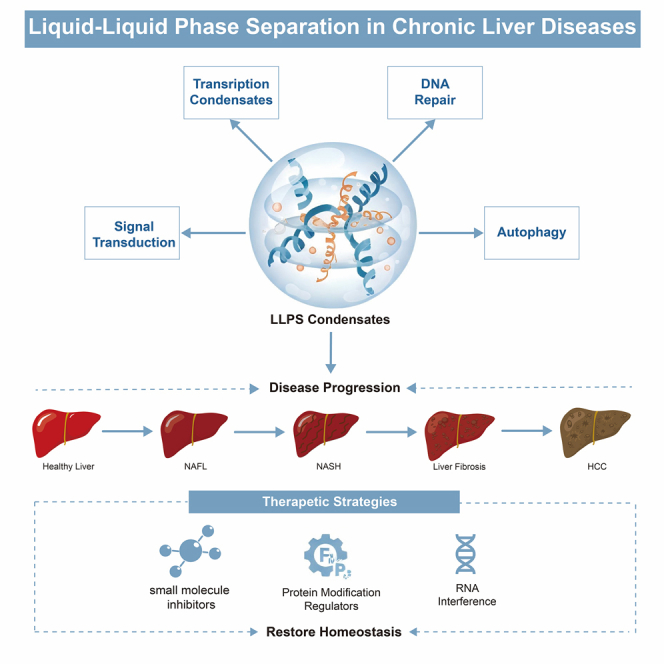

## Introduction

Chronic liver diseases (CLDs) represent a major global health burden, encompassing a range of liver disorders persisting for over six months due to various etiologies. These include non-alcoholic fatty liver disease (NAFLD), viral hepatitis, liver fibrosis, and hepatocellular carcinoma (HCC).^1^ Among these, NAFLD is one of the most prevalent CLDs worldwide, with incidence rates rising in parallel with the global epidemic of obesity and metabolic syndrome.^2^^,^^3^^,^^4^^,^^5^ Research has identified a strong correlation between NAFLD and both liver fibrosis and HCC.^6^^,^^7^^,^^8^ It typically begins as non-alcoholic fatty liver (NAFL), which, if left unaddressed, can progress to non-alcoholic steatohepatitis (NASH), accompanied by hepatocyte injury and inflammatory responses.^9^ NASH can further lead to liver fibrosis, characterized by the formation of scar tissue within the liver, which in severe cases may progress to cirrhosis.^10^^,^^11^ Liver fibrosis plays a pivotal role in the transition from NASH to HCC, as increasing fibrosis severity is associated with a higher likelihood of progression to HCC in some patients.^12^ Consequently, NAFLD represents a progressively worsening disease spectrum, where liver fibrosis serves as both a critical intermediate stage and a severe late-stage complication facilitating the development of HCC. Given its significant implications and the rising global public health burden, there is an urgent need to advance research and implementation of early diagnostic and effective therapeutic strategies for NAFLD to mitigate the risk of liver-related complications. A deeper understanding of the pathological mechanisms underlying NAFLD is essential for the prevention and treatment of liver fibrosis and HCC.

Intracellular liquid–liquid phase separation (LLPS) has emerged as a novel organizational mechanism for biomolecules, gaining significant attention in recent research. LLPS refers to the process in which biomacromolecules, such as proteins and RNAs, condense into structured aggregates at the nanoscale and separate into distinct phases within cells.^13^ By forming functional biomolecular condensates, LLPS enables efficient regulation and dynamic cellular responses, playing a critical role in maintaining cellular functions and contributing to disease pathogenesis.^14^^,^^15^ Current studies reveal that, in addition to membrane-bound organelles, eukaryotic cells also contain membraneless biomolecular condensates composed of organized proteins and nucleic acids. This phenomenon is proposed to represent a potential mechanism for the formation of highly specific and spatiotemporally regulated signaling structures within cells via LLPS.^16^ Unlike classical membrane-bound organelles such as the endoplasmic reticulum and Golgi apparatus, LLPS mediates reversible and dynamic interactions between proteins or between proteins and RNAs. This mechanism underpins the formation of membraneless organelles, including nucleoli, stress granules, Cajal bodies, P granules, promyelocytic leukemia (PML) bodies, and cytoplasmic DNA sensor protein cyclic GMP-AMP synthase (cGAS).^17^^,^^18^^,^^19^^,^^20^^,^^21^^,^^22^ Additionally, LLPS plays a pivotal role in regulating gene expression, signal transduction, cellular stress responses, and cell cycle control.^23^ Through LLPS, cells can rapidly respond to environmental changes by modulating protein concentration, activating signaling pathways, and concentrating functional proteins at specific sites, thereby achieving precise control of cellular functions.

In recent years, the critical role of LLPS in CLDs has garnered widespread attention. Dysregulated LLPS is considered a potential driver of the pathological progression of NAFLD and related liver disorders by influencing the assembly of protein complexes and signaling networks.^24^ LLPS modulates the functions of tumor suppressors such as p53, thereby affecting HCC cell proliferation, migration, and drug resistance.^25^ Moreover, LLPS is involved in regulating hepatic metabolism and inflammatory responses, exacerbating the progression of liver diseases.^26^^,^^27^ Consequently, LLPS-mediated molecular mechanisms play a pivotal regulatory role in the malignant transition from NAFLD to liver fibrosis and HCC. This review focuses on the formation mechanisms of LLPS and its cellular biological functions, combining current literature and research progress. It specifically explores the key role of LLPS in the progression of NAFLD, with a particular emphasis on the molecular mechanisms involved in liver fibrosis and HCC transformation. By analyzing the LLPS-mediated signaling pathways and metabolic regulation, we aim to elucidate its core impact on the evolution of NAFLD-associated liver diseases in chronic liver disease. It should be noted that most current LLPS studies are based on *in vitro* models or gene-manipulated animal experiments, and direct evidence from human liver disease tissues remains limited. Many conclusions are derived from correlation analyses, and the dynamic process of phase separation is difficult to track in real-time in the *in vivo* liver microenvironment. Therefore, whether LLPS is a driving factor or a pathological concomitant phenomenon still requires further time- and space-resolved studies for validation.

## Biological characteristics of liquid-liquid phase separation

### Fundamental physical principles of liquid-liquid phase separation

**Figure 1:**
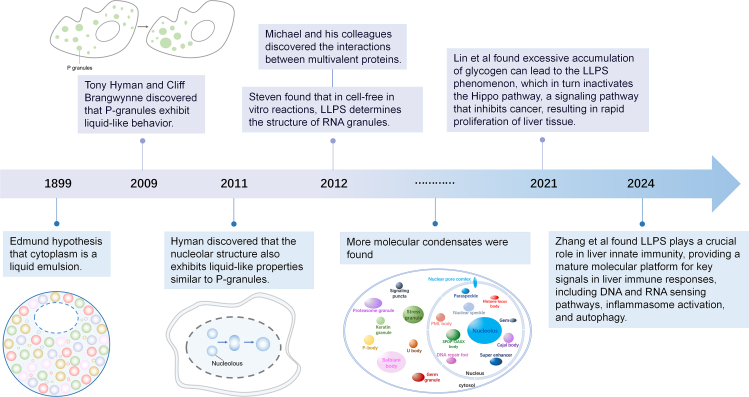
History of the discovery and development of LLPS Representative milestone findings promote the development of LLPS are enumerated in the figure.

As shown in Figure 1, the study of LLPS has a long history. As early as 1899, Edmund Beecher Wilson proposed that the cytoplasm might contain “mixtures of multiple liquids,” suggesting the presence of “suspended droplets with distinct chemical properties.”^28^ In 2009, Brangwynne observed liquid-like P granules in the embryos of Caenorhabditis elegans, sparking renewed interest in LLPS as a fundamental cellular phenomenon.^20^ To better understand the driving force behind LLPS, the Flory-Huggins theory offers an explanation. This theory suggests that LLPS is a balance between the mixing entropy and the energy interactions between the polymer and the solvent.^29^ In simple terms, when the attractive forces between biomolecules in a solution are sufficiently strong and their concentration reaches a certain threshold, the system spontaneously forms a concentrated phase enriched with the biomolecules to reduce the overall free energy.^15^ This process is essentially a balance between entropy reduction and energy minimization, similar to the phenomenon of oil-water separation, where the system tends to form a two-phase state with lower energy. A key feature of LLPS is the existence of a threshold concentration. When the concentration exceeds this threshold, phase separation occurs, resulting in a dilute solution phase and a concentrated phase rich in biomolecules.^30^ In recent years, LLPS has been shown to play a critical role in the progression of chronic liver diseases. Lipid droplets, intracellular organelles that store neutral lipids, are essential for lipid homeostasis and are implicated in disease processes.^29^ Li et al. demonstrated that the lipid droplet fusion protein Cidec can enrich at lipid droplet-droplet contact sites via a gel-like phase separation mechanism. This process facilitates the assembly of fusion discs that allow lipid exchange and transfer between droplets, promoting the growth of larger lipid droplets and efficient lipid storage.^31^ Therefore, exploring the functional roles of LLPS in chronic liver diseases is crucial for uncovering underlying pathological mechanisms.

### Formation and regulation of biomolecular condensates

#### Driving forces behind biomolecular condensate formation

**Figure 2:**
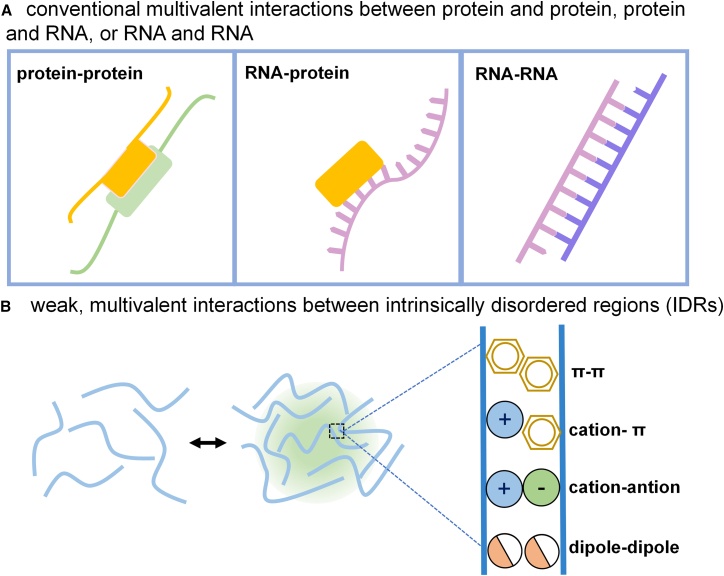
Multivalent interactions between molecules in LLPS (A) One type is conventional multivalent interactions, including protein-protein, RNA-protein, and RNA-RNA interactions mediated by base pairing or protein bridging, which provide the fundamental dynamics for LLPS. (B) The other type involves weak multivalent interactions between intrinsically disordered regions (IDRs), primarily including π-π interactions, cation-π interactions, cation-anion interactions, and dipole-dipole interactions. These weak interactions play a critical role in driving the formation of droplet-like condensates by IDRs and maintaining their dynamic properties.

Proteins, DNA, and RNA are the primary components and mediators driving LLPS, interacting to create highly multicomponent systems.^32^^,^^33^ Phase separation is typically driven by weak interactions between amino acid residues and other macromolecules. These weak interactions primarily include π-stacking, electrostatic forces, cation-π interactions, and hydrophobic contacts.^34^^,^^35^ Proteins capable of undergoing phase separation often contain intrinsically disordered regions (IDRs) and low-complexity regions (LCRs), which are widely recognized as key drivers and regulators of biomolecular condensates.^19^^,^^36^ IDRs are enriched in specific amino acids, including aromatic residues, charged residues, or hydrophilic residues. Aromatic residues such as phenylalanine, tyrosine, and tryptophan stack to form π-electron clouds (π-π interactions) or interact with positively charged residues via cation-π interactions.^37^ Hydrophilic residues such as serine, glutamine, glutamate, arginine, and lysine enable electrostatic interactions, further promoting LLPS.^38^ IDRs can also be classified into subtypes such as RG/RGG domains, FG domains, and prion-like domains, each participating in various non-covalent interactions responsible for driving phase transitions.^39^^,^^40^^,^^41^ Furthermore, LCRs, due to their lack of stable tertiary structures and their flexible conformations, are particularly conducive to LLPS formation.^42^ In addition to protein-centered interactions, RNA molecules themselves can serve as scaffolds for condensate formation through multivalent RNA-RNA interactions.^43^ These interactions are primarily mediated by base complementary pairing, allowing RNA chains to form reversible weak bond networks.^44^ This principle is exemplified in the assembly of various ribonucleoprotein particles, where specific RNA sequences or structures promote phase separation through intermolecular interactions.^17^ Furthermore, RNA can act as a platform to recruit RNA-binding proteins, effectively increasing local valency and facilitating the formation of complex multiphase condensates (Figure 2).^45^

#### Regulation of biomolecular condensate formation

The formation of biomolecular condensates is governed by a variety of factors. Reversible LLPS is highly sensitive to environmental conditions that influence multivalent interactions. The intracellular environment is the core site for LLPS to occur and be regulated. When the concentration of specific proteins or RNA molecules exceeds the solubility threshold, they undergo LLPS, transitioning from a solution to droplets. As the concentration increases, the weak interactions between proteins and RNA are amplified, further promoting the formation of these “droplets”.^46^ At the same time, phase separation is also regulated by various physiological and pathological factors. Fluctuations in homeostasis directly affect the interactions between biomolecules, thereby influencing the phase separation behavior of biomolecules *in vivo*, leading to their aggregation and disaggregation.^47^ For example, temperature changes alter the hydrophobicity of proteins and the strength of weak interactions^48^; changes in pH can protonate or deprotonate key residues (such as histidine, aspartic acid, and glutamic acid), thereby altering charge distribution and electrostatic interactions—factors that are crucial for many LLPS drivers.^49^ Ionic strength can shield electrostatic attraction or repulsion; high salt concentrations can dissolve aggregates stabilized by opposite charges, while low salt concentrations may promote their formation.^50^ Osmotic pressure and macromolecular crowding affect the effective concentration and excluded volume inside the cell, which can lower the concentration threshold required for phase separation to occur.^51^ Outside the cell, the stiffness of the extracellular matrix (ECM) can influence the mechanical signal transduction of the cell through receptors such as integrins. This activation may lead to cytoskeletal remodeling and an increase in intracellular tension, thereby promoting the formation of certain aggregates involved in pro-fibrotic or proliferative signaling.^52^^,^^53^ In contrast, a softer microenvironment may inhibit such aggregation. Therefore, LLPS is not a static phenomenon, but a highly responsive process that allows the cell to sense and adapt to its constantly changing internal and external environment. The dysregulation of these environmental signals can lead to abnormal phase separation, promoting disease progression. As shown in Table 1, various environmental factors affect LLPS behavior.

**Table 1:** Impact of environmental factors on LLPS behavior

Factors	Effect on LLPS	Proposed Mechanism	Reference
Protein Concentration	Higher protein concentration promotes LLPS	A higher local concentration of proteins increases the likelihood of phase separation, as the proteins can interact more readily to form condensates.	Weber and Brangwynne^46^
Temperature	High temperatures favor LLPS; low temperatures inhibit LLPS	Temperature affects molecular motion and protein-protein interactions. Increased temperature often increases molecular collisions, encouraging LLPS, while cold reduces it.	Ren et al.^48^
pH	Acidic pH promotes LLPS; Alkaline pH inhibits LLPS	Low pH can facilitate the phase separation of certain proteins by altering charge distribution and electrostatic interactions. Higher pH can disrupt such interactions, preventing LLPS.	Hughes^49^
Salt Concentration	Higher salt concentrations can disrupt LLPS	Salt ions can shield electrostatic interactions between proteins, decreasing their ability to undergo LLPS.	Sternke-Hoffmann et al.^50^
Crowding Effect	Promotes LLPS	Increases effective concentration, reduces solvent availability	Priyanka et al.^51^
ECM Stiffness	Stiffer ECM promotes LLPS	A stiffer ECM increases mechanical tension in cells, which can facilitate LLPS by altering cytoskeletal dynamics and protein concentration.	Horii et al.^52^; Tiskratok et al.^53^

Post-translational modifications (PTMs) of proteins are critical regulatory elements of LLPS.^54^ Modifications such as phosphorylation, acetylation, ubiquitination, and methylation regulate biomolecular phase separation by altering the strength of intermolecular interactions or directly modifying the valency of interacting molecules.^55^^,^^56^^,^^57^^,^^58^ For instance, arginine methylation can inhibit LLPS by weakening protein-protein interactions. Such modifications suppress the LLPS of proteins such as Ddx4, FUS, and FMRP.^59^^,^^60^^,^^61^ Conversely, other PTMs, such as phosphorylation, may exert opposing effects on LLPS in specific systems. Hearst et al. reported that the phosphorylation of Coilin, a marker protein of Cajal bodies, is associated with their formation.^55^ Additionally, post-transcriptional modifications also regulate LLPS behavior.^62^ RNA N6-methyladenosine (m^6^A) is among the most common RNA modifications. Wang et al. demonstrated that the m^6^A reader protein YTHDF2 exhibits LLPS properties, with its phase separation capability significantly enhanced upon binding to m^6^A-modified mRNA.^63^ These findings highlight the diversity and complexity of PTMs and post-transcriptional modifications in LLPS regulation, providing new insights into the formation and function of dynamic intracellular structures.

## Regulation of liver physiology by liquid-liquid phase separation

### Liquid-liquid phase separation and intracellular signal transduction

**Figure 3:**
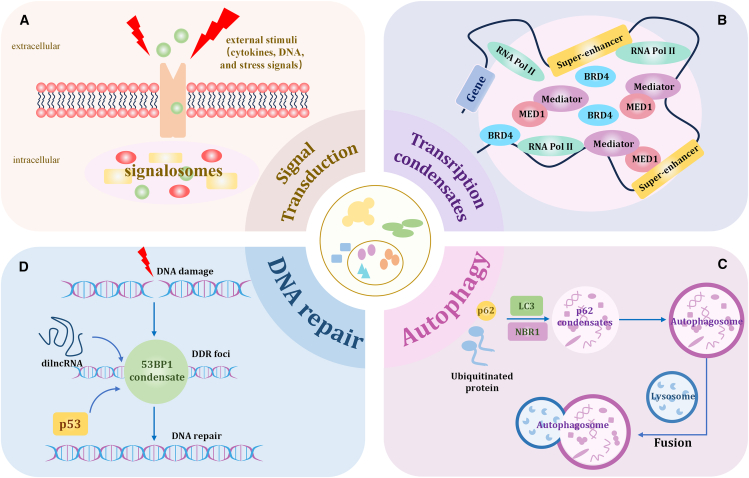
Functions of biomolecular condensates (A) LLPS in signal transduction. LLPS promotes the assembly of intracellular signaling complexes (signalosomes) upon external stimulation. (B) LLPS in transcriptional regulation. Transcription condensates formed by LLPS, including factors such as RNA Pol II, BRD4, and mediator, play a role in the regulation of gene expression. (C) LLPS in autophagy. p62-mediated condensates facilitate the encapsulation of ubiquitinated proteins by recruiting NBR1 and LC3, ultimately forming autophagosomes that fuse with lysosomes to complete degradation. (D) LLPS in DNA damage repair. LLPS drives the formation of 53BP1 condensates, which cooperate with dilncRNA and p53 to promote the assembly of DDR foci and initiate DNA repair. These functions highlight the indispensable role of LLPS in critical cellular biological processes. RNA Pol II, RNA polymerase II; BRD4, bromodomain containing 4; DDR, DNA damage response; 53BP1, P53 binding protein 1; NBR1, next to BRCA1 gene 1; LC3, microtubule-associated protein 1A/1B light chain 3.

Signal transduction refers to the intracellular process by which cells convert extracellular stimuli (such as cytokines, DNA, and stress signals) into specific biological responses. LLPS facilitates the formation of membraneless signaling complexes (“signalosomes”) and plays a central role in regulating the spatiotemporal dynamics of various critical signaling pathways (Figure 3A).^64^

#### cGAS-STING signaling pathway

The cGAS-STING signaling pathway is a crucial component of the mammalian innate immune system and is implicated in various diseases, including inflammation, infections, autoimmune disorders, metabolic dysfunction, and cancer.^65^ LLPS has been proven to be a key mechanism for the efficient transmission and amplification of signal cascades. In response to stimuli such as pathogens or damaged cytoplasmic dsDNA, cGAS, as a cytosolic DNA sensor, undergoes LLPS, which is a core step in the activation of this pathway. The N-terminal disordered region of cGAS, rich in positively charged amino acids, interacts electrostatically with the negatively charged backbone of dsDNA, while the interactions at DNA binding sites A, B, and C in the C-terminal catalytic domain further stabilize this complex.^66^ In the concentrated microenvironment formed by phase separation, cGAS dimerizes and forms micron-sized droplets, significantly enhancing its enzymatic activity and catalyzing the formation of the secondary messenger cGAMP from ATP and GTP.^22^ Subsequently, cGAMP binds to the STING receptor on the endoplasmic reticulum (ER), inducing STING’s migration from the ER to the ER-Golgi intermediate compartment (ERGIC) and the Golgi, where LLPS occurs, forming a signaling complex that includes TBK1 (TANK-binding kinase 1) and IRF3 (interferon regulatory factor 3).^67^ This activation of STING then triggers a series of downstream signaling events, promoting the expression of type I interferons and interferon-stimulated genes.^68^ Additionally, STING can recruit and activate key components of the NF-κB pathway via LLPS, further enhancing the production of pro-inflammatory cytokines in collaboration with activated NF-κB. In the context of chronic liver diseases, the abnormal activation of this pathway serves as a core bridge linking hepatocyte damage, inflammation, and fibrosis.^69^ Metabolic stress and lipid overload exacerbate DNA damage and upregulate cGAS expression, thereby enhancing its tendency to undergo phase separation.^70^ Moreover, inflammatory mediators such as TNF-α, IL-6, and the continuous activation of the NF-κB pathway further reinforce STING-dependent LLPS through positive feedback mechanisms.^71^

#### Nuclear factor kappa-B signaling pathway

Nuclear factor kappa-B (NF-κB) is a protein complex and an important nuclear transcription factor within the cell. For a long time, it has been regarded as a typical pro-inflammatory signaling pathway and is a central regulator of responses related to inflammation, immunity, and cell survival.^72^ The NF-κB family comprises several members, including IKKα, IKKβ, and NEMO (also known as IKKγ), which typically exist as dimers in an inactive state. Upon stimulation by pro-inflammatory signals, NF-κB dimers are released and translocate into the nucleus, where they participate in gene expression.^73^ LLPS appears to be a key step in NF-κB signal transduction:NEMO, as a scaffold protein, can specifically recognize and bind various types of ubiquitin chains. This binding is not a simple one-to-one interaction, but rather, through its own multivalency and the multivalency of the ubiquitin chains, drives the formation of a dynamic, droplet-like biomolecular condensate.^74^ In the concentrated microenvironment formed by phase separation, NEMO recruits and concentrates the IKKα and IKKβ kinases, significantly promoting their *trans*-autophosphorylation and activation.^74^ The activated IKK complex subsequently phosphorylates IκBα, leading to its ubiquitination and degradation, thereby releasing the inhibited NF-κB dimers, which translocate to the nucleus and initiate the transcriptional programs of numerous inflammatory and pro-survival genes.^75^ Therefore, LLPS provides a highly organized reaction center for the NF-κB signaling pathway, ensuring that inflammatory signals are rapidly, efficiently, and controllably amplified.

#### Hippo signaling pathway

The Hippo signaling pathway is an evolutionarily conserved network that regulates various biological processes by controlling the expression of key target genes.^76^ At its core lies a kinase cascade composed of serine/threonine protein kinases that modulate cell proliferation and apoptosis by phosphorylating downstream effectors, Yes-associated protein (YAP) and transcriptional coactivator with PDZ-binding motif (TAZ).^76^ The Hippo pathway, a crucial regulatory axis controlling cell proliferation, apoptosis, and organ size, is significantly influenced by the mechanisms of LLPS in regulating the subcellular localization and activity of its core transcriptional co-activators, YAP/TAZ. Studies have demonstrated that TEAD proteins can undergo LLPS to form condensates, which serve as molecular hubs to efficiently recruit YAP/TAZ client proteins.^77^ This recruitment plays a crucial role in regulating the subcellular localization and transcriptional activity of YAP/TAZ. Furthermore, O-GlcNAcylation of YAP has been shown to promote its LLPS behavior, thereby facilitating its nuclear translocation and enhancing its transcriptional activation potential.^78^

In addition, upstream regulatory factors of the Hippo pathway, such as MST1/2 and LATS1/2, may also undergo dynamic assembly through LLPS-related mechanisms, modulating the activity and spatiotemporal distribution of kinase complexes.^79^ Simultaneously, upstream regulators such as AMOT, KIBRA, and SLMAP have been found to form functional antagonistic dynamic condensates via LLPS and phase separation, which, depending on the context, can positively or negatively regulate the kinase activity of the Hippo pathway, further enriching the spatial and functional complexity of this signaling axis.^80^

#### Cyclic adenosine monophosphate signaling pathway

The protein kinase A (PKA) signaling pathway is a key transduction cascade in eukaryotic cells that responds to extracellular stimuli such as hormones to regulate intracellular cyclic adenosine monophosphate (cAMP) levels and the phosphorylation status of downstream substrate proteins.^81^ Through this mechanism, PKA finely modulates a wide range of biological processes, including metabolism, cell proliferation, differentiation, and gene expression.^82^ It is noteworthy that the phase separation of its regulatory subunit RIα plays a crucial role in cellular signal transduction, particularly in regulating the activity and spatial localization of PKA.^83^ RIα is rich in IDRs and LCDs, which endow it with the potential to undergo LLPS. Studies have shown that the RIα can respond to changes in cAMP levels by undergoing LLPS, forming condensates with reversible aggregation.^84^ This phase separation phenomenon not only contributes to the compartmentalization of cAMP signaling but also facilitates the creation of localized high-concentration zones of cAMP and PKA activity, enabling precise temporal and spatial regulation of signaling. In the context of chronic liver diseases, sustained hepatic injury and stress may disrupt the phase separation behavior of the regulatory subunit, leading to aberrant PKA activation. This, in turn, promotes excessive hepatocyte proliferation and fibrosis, exacerbating the pathological conditions of the liver.

### Liquid-liquid phase separation in transcriptional regulation

Transcription is the first step in gene expression and is a fundamental process for protein synthesis. This process is tightly regulated by complex mechanisms to ensure precise gene expression. LLPS plays a crucial role in transcriptional regulation, and its dysregulation can lead to the development of various diseases, particularly HCC (Figure 3B).

#### RNA-mediated liquid-liquid phase separation in transcriptional regulation and nuclear function

LLPS is a critical mechanism regulating various intracellular biological processes, including transcription, RNA splicing, and processing, all of which are essential nuclear activities.^43^ RNA, as a central regulator of LLPS, directly participates in these processes by modulating their dynamic behavior and spatial organization. By orchestrating the distribution and interaction of molecules, RNA enables precise control of gene expression.^43^ During transcription, RNA polymerase II (Pol II) features a C-terminal disordered domain (CTD) whose flexibility allows it to interact with RNA-binding proteins, such as FUS.^85^ These interactions form transient networks via LLPS, resulting in functional condensates that provide a spatially and chemically optimized environment for transcription initiation and RNA processing. This facilitates the recruitment of RNA processing factors and the regulation of gene expression.^86^ CTD-mediated phase separation is thus crucial for maintaining nuclear dynamic homeostasis and fine-tuning gene expression. Experimental findings have highlighted the interaction between Pol II CTD and FUS, demonstrating its essential role in transcriptional regulation. Furthermore, Pol II CTD has been shown to spontaneously form droplets *in vitro*, enhancing its organizational role in gene expression.^87^ This droplet-forming behavior arises from its intrinsic sequence properties and is further modulated by interacting proteins and nucleic acids. For instance, the binding of FUS to Pol II CTD alters the physical properties of these droplets, thereby regulating the recruitment of transcription factors and the assembly of RNA processing complexes. In vitro experiments have revealed the reversible nature of these droplets, indicating their dynamic responsiveness to physiological and pathological signals within the cellular environment. Collectively, these findings highlight the pivotal role of LLPS in driving gene transcription and RNA-related processes, offering deeper insights into the complex biological activities within the nucleus.

#### Critical role of liquid-liquid phase separation in transcriptional mechanisms during non-alcoholic fatty liver disease progression

In certain cancer types, tumor-associated fusion proteins such as FET-ETS and FUS exhibit significant LLPS activity. These proteins interact with Pol II to form biomolecular condensates, reshaping transcriptional regulatory networks and leading to aberrant gene expression.^88^ This dysregulated phase separation has been recognized as a crucial pathogenic mechanism in cancer progression. Similarly, LLPS mechanisms play a key role in the progression of chronic liver diseases. In NAFLD, RNA-binding proteins regulate the expression of metabolism-related genes through LLPS, affecting lipid metabolism and energy balance, thereby accelerating disease progression. For example, BRD4 forms transcriptional activation condensates at super-enhancer regions via LLPS,^89^^,^^90^ driving the expression of genes associated with inflammation and lipid metabolism, thereby promoting the development of NAFLD. As NAFLD worsens, it can advance to more severe pathological states, including liver fibrosis and HCC. These pathological transitions are tightly linked to LLPS mechanisms. During liver fibrosis, mediator complex subunit MED1 and epigenetic regulator BRD4 exhibit prominent LLPS activity, regulating the expression of fibrotic genes and ECM production.^91^ This process involves dynamic enhancer-promoter interactions, driving the overexpression of fibrosis-related genes. Fibrosis not only promotes chronic inflammation and excessive ECM deposition through LLPS mechanisms but also establishes a microenvironment conducive to the transition from NAFLD to liver cancer. In HCC, LLPS drivers such as BRD4 and FUS form transcriptional condensates to regulate gene expression in cancer cells, promoting cell proliferation, remodeling of the tumor microenvironment, and enhanced cell survival. Studies have shown that BET inhibitors, such as JQ1, can disrupt BRD4-Med1 condensates, demonstrating potential therapeutic effects in the treatment of liver fibrosis and HCC.^92^

### Liquid-liquid phase separation in autophagy

Autophagy is a critical cellular process in which damaged or abnormal organelles, macromolecules, or protein aggregates are transported to lysosomes for degradation through double-membrane vesicles. This process is essential for maintaining cellular homeostasis and recycling intracellular materials.^93^ LLPS has been demonstrated to play a significant role in the regulation of autophagy. It not only participates in the assembly of autophagosome formation sites but also contributes to various steps, such as protein sorting and delivery, as well as the regulation of signaling pathways. As such, LLPS has emerged as a focal point in research into cellular biology and disease mechanisms (Figure 3C).

#### The relationship between liquid-liquid phase separation and autophagosome formation

LLPS plays a critical role in the assembly of autophagosome formation sites. The pre-autophagosomal structure (PAS) is a liquid-like biomolecular condensate formed by Atg proteins, with the Atg1 complex—comprising Atg1, Atg13, Atg17, Atg29, and Atg31—serving as the core component of this process.^94^ These proteins are rich in IDRs, which enable them to aggregate via LLPS mechanisms, forming droplet-like condensates. Studies have shown that the Atg1 complex undergoes phase separation *in vitro*, generating droplet-like structures. *In vivo*, these Atg1 droplets interact with membrane structures through specific protein-protein interactions, facilitating autophagosome formation.^94^ LLPS is thus considered a key mechanism driving the assembly of autophagosome formation sites in higher eukaryotes. The IDRs within Atg proteins are regarded as the central drivers of LLPS, promoting protein aggregation through multivalent interactions and forming droplet-like condensates in the cytoplasm.^95^ Key proteins in the Atg1 complex, such as Atg17, also form condensates via LLPS, exhibiting dynamic behavior both *in vitro* and *in vivo*.^94^ These condensates interact with membrane-associated Atg proteins, such as Atg9, via specific protein-protein interactions, serving as nucleation points for autophagosome membrane expansion.^96^ Similarly, Atg8, a critical regulator of autophagosome formation, collaborates with Atg3 to facilitate the LLPS process, forming more stable droplets within PAS.^97^ This phenomenon has been observed in both Saccharomyces cerevisiae and mammalian cells, indicating the conserved nature of LLPS in autophagy mechanisms across species. Specifically, experiments have shown that Atg8 undergoes spontaneous LLPS through its IDRs and membrane anchoring ability, recruiting other Atg proteins and maintaining active zones for autophagosome formation.^98^ Furthermore, under conditions of nutrient deprivation or cellular stress, LLPS-formed condensates rapidly assemble and disassemble, modulating the activity of Atg proteins.^99^^,^^100^ This highlights the critical role of LLPS in enabling cells to adapt quickly to environmental changes. Additional studies have demonstrated that mutant Atg1 complexes lacking IDRs are unable to undergo effective LLPS, thereby failing to form functional PAS in cells.^101^ These findings suggest that LLPS not only serves as the physical driving force for Atg protein aggregation but is also essential for the realization of their biological functions. In summary, LLPS facilitates the orderly aggregation and spatial localization of specific Atg proteins, providing both structural and functional foundations for autophagosome formation.

#### Role of liquid-liquid phase separation in misfolded protein processing

The aggregation of misfolded proteins within cells is a hallmark of various degenerative and metabolic diseases,^102^^,^^103^^,^^104^ LLPS plays a pivotal role in managing this process. Misfolded proteins are often ubiquitinated and subsequently recognized by the scaffold protein p62 (also known as SQSTM1).^105^ Through its ubiquitin-associated (UBA) domain, p62 specifically binds ubiquitinated misfolded proteins and employs LLPS mechanisms to concentrate these proteins into droplet-like biomolecular condensates. These condensates are then targeted for degradation via selective autophagy.^106^ LLPS effectively clusters misfolded proteins, preventing their random diffusion in the cytoplasm. p62 further facilitates the aggregation of these proteins into organized droplet-like condensates using its distinct domains, such as the PB1 domain. This ordered condensation allows misfolded proteins to be efficiently recognized and degraded by autophagosomes, mitigating the toxic effects of protein aggregates within the cell.^107^ Beyond regulating protein aggregation, LLPS enhances the interactions between p62 and autophagy-related proteins. For instance, p62 binds to LC3, expediting the recognition and clearance of misfolded protein aggregates. This LLPS-mediated process assigns p62 a “filtering” role, enabling it to rapidly concentrate and deliver potentially harmful proteins to autophagosomes, thereby maintaining intracellular homeostasis.^108^ This mechanism is particularly critical in cells with high levels of misfolded protein accumulation. By reducing the toxicity of aggregates, p62 helps minimize protein aggregation-related cellular damage and promotes the maintenance of cellular homeostasis.

In the context of CLD, many key proteins that are prone to misfolding also play crucial roles in disease progression. Misfolded proteins, including those associated with fibrosis, liver inflammation, and HCC, aggregate and disrupt liver function. LLPS may help regulate these proteins by facilitating their degradation, thereby alleviating the damage caused by protein accumulation. Table 2 later in discussion summarizes specific proteins involved in CLD progression and their mechanisms of action.

**Table 2:** Specific proteins involved in CLD progression and their mechanisms

Protein	Role in CLD Progression	Mechanism of Action	Reference
α1-antitrypsin (AAT)	Deficiency linked to liver damage	Misfolding of AAT in the liver leads to the accumulation of misfolded proteins, triggering inflammation and fibrosis in CLD.	Marzi et al.^109^
HFE (Hemochromatosis gene)	Iron overload, contributing to liver injury	Mutations cause excess iron accumulation, leading to oxidative stress and hepatocyte damage.	Kouroumalis et al.^110^
Fibronectin	Extracellular matrix remodeling	Misfolding of fibronectin can disrupt normal matrix function, contributing to liver fibrosis and cirrhosis.	Wu et al.^111^
Glypican-3 (GPC3)	Tumor suppressor involved in liver carcinoma	Misfolding or abnormal expression in hepatocytes can contribute to HCC in the context of CLD.	Devan et al.^112^
P53	Tumor suppressor protein, mutated in many cancers	Mutations or misfolding lead to impaired cell cycle regulation and apoptosis, contributing to hepatocellular carcinoma in CLD progression.	Petronilho et al.^25^
Collagen Type I	Fibrosis marker	Abnormal collagen deposition due to liver injury promotes fibrosis in chronic liver diseases such as cirrhosis.	Khurana et al.^113^

As shown above, misfolded proteins such as AAT and ceruloplasmin contribute directly to liver toxicity due to their impaired degradation and accumulation.^109^^,^^110^ In addition, fibronectin and type I collagen are key factors in liver fibrosis. Their abnormal deposition can be alleviated by regulating their synthesis and secretion pathways, thereby reducing excessive ECM accumulation. Future studies may explore the potential role of LLPS in this process.^111^^,^^113^ Oncogenic proteins such as p53 and GPC3, which are often misregulated in hepatocellular carcinoma, are also candidates for LLPS-mediated degradation, highlighting a broader regulatory role of LLPS in CLD-related carcinogenesis.^25^^,^^112^

### Liquid-liquid phase separation and DNA damage repair

Genomic DNA within cells is continuously exposed to damage caused by exogenous factors such as ultraviolet radiation, ionizing radiation, and radioactive substances, as well as endogenous agents such as reactive oxygen species (ROS).^114^ To maintain genomic stability, organisms have evolved a sophisticated and precise DNA damage repair system. This system encompasses key processes such as damage signaling, repair mechanisms, and the induction of cell death.^115^ Recent studies have highlighted the emerging role of LLPS in DNA damage repair (Figure 3D).^116^

#### Liquid-liquid phase separation: A facilitator of DNA damage sensing and protein recruitment

NAFLD is often accompanied by disrupted lipid metabolism and oxidative stress in the liver. Excessive fat accumulation promotes the production of ROS, an endogenous factor that causes genomic DNA damage.^117^ LLPS plays a critical role in the early stages of DNA damage repair by aiding in the rapid recognition of damage sites and amplifying damage signals. Upon DNA damage, sensor proteins such as the MRN complex and PARP1 quickly recognize and bind to the damage sites.^118^ Chappidi et al. reported that PARP1, a key early DNA damage sensor, is crucial for detecting DNA double-strand breaks.^119^ When activated, PARP1 induces PARylation modifications, which promote the formation of condensates containing other DNA repair-related proteins, initiating the repair process and preventing detrimental outcomes such as mutations due to unrepaired damage. This mechanism is critical for maintaining genomic stability in hepatocytes during the early stages of NAFLD. Similarly, DNA damage activates PARP1, along with FUS/TLS, EWS, and TAF15, which undergo phase separation at the damage sites.^120^ The formation of condensates further facilitates the local enrichment of repair proteins. Kilic et al. demonstrated that 53BP1 forms condensates via LLPS at DNA damage sites, concentrating repair proteins and signaling factors such as DNA polymerases and ligases.^121^ These enriched repair proteins precisely address damaged DNA, such as oxidative stress-induced base lesions and single-strand breaks, alleviating the cellular stress caused by persistent damage and, to some extent, slowing the progression of liver fibrosis while maintaining basic liver tissue function. Pessina et al. further revealed that DNA double-strand breaks (DSBs) induce the assembly of transcriptional activators, driving RNA synthesis and promoting the phase separation of DDR protein 53BP1, thereby regulating the DNA repair process.^122^ These findings provide new perspectives on the role of LLPS in DNA damage repair and genomic stability.

#### Liquid-liquid phase separation: A critical hub for the dynamic regulation of repair complexes

As the DNA damage repair process progresses, LLPS continues to exert a significant influence. HCC represents the terminal stage of liver disease progression. During the development of HCC, factors such as viral infections (e.g., hepatitis B virus and hepatitis C virus), genetic mutations, and prolonged inflammatory microenvironments contribute to severe DNA damage, including complex forms such as double-strand breaks (DSBs).^123^ LLPS facilitates the dynamic assembly and disassembly of repair complexes throughout this process. In DSB repair, protein-protein and protein-nucleic acid interactions are essential for forming stable repair complexes and ensuring efficient repair responses. LLPS enables the precise spatiotemporal regulation of these interactions, allowing repair factors to rapidly assemble into functional complexes within condensates based on cellular demands. Upon the completion of the repair, these condensates disassemble in a timely manner.^124^^,^^125^ In HCC cells, however, LLPS mechanisms may be altered due to aberrant regulatory processes in tumor cells. This alteration could represent an adaptive strategy by tumor cells to cope with high-frequency DNA damage or a potential therapeutic target. LLPS-mediated dynamic regulation of repair complexes ensures that DNA repair mechanisms operate with efficiency and accuracy under varying levels of damage. This dynamic control helps liver cells maintain genomic stability in complex pathological environments, slowing the progression of liver disease. It provides crucial support for the survival and function of hepatocytes, enabling the orderly progression of DNA repair processes and preserving normal cellular and tissue activities in the liver. Recent studies have further revealed the unique role of the translation elongation factor EEF1E1 in HCC, where it forms condensates within the cell through LLPS, activating the PTEN/AKT signaling pathway and significantly enhancing the DNA damage repair capacity of hepatocellular carcinoma cells. This, in turn, provides cancer cells with a stronger survival advantage and stemness characteristics.^126^ This finding not only highlights EEF1E1 as a critical node linking LLPS and the DNA repair network, but also suggests that it may serve as a key molecule driving the malignant progression of HCC.

## Liquid-liquid phase separation in chronic liver diseases

**Figure 4:**
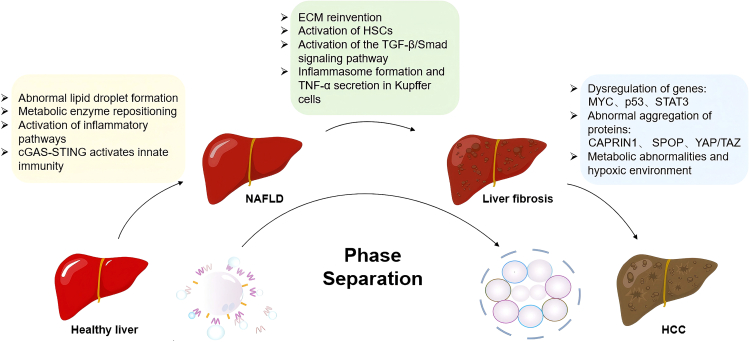
Role of liquid-liquid phase separation in liver diseases Through the mechanism of phase separation, distinct molecular, and cellular biological changes are observed at different stages: abnormal lipid droplet formation, metabolic enzyme repositioning, activation of inflammatory pathways, and cGAS-STING-mediated immune activation drive the transition from a healthy liver to NAFLD. In the liver fibrosis stage, ECM remodeling, HSCs activation, enhancement of the TGF-β/Smad signaling pathway, and increased inflammasome formation and TNF-α secretion are observed. Finally, in HCC, the dysregulation of genes such as MYC, p53, and STAT3, as well as the abnormal aggregation of proteins such as CAPRIN1, SPOP, and YAP/TAZ, occur alongside metabolic abnormalities and hypoxia-induced angiogenesis. These pathological processes mediated by LLPS reveal the molecular mechanisms underlying liver disease progression, providing potential therapeutic targets. cGAS, cyclic GMP-AMP synthase; STING, stimulator of interferon genes; ECM, extracellular matrix; HSCs, hepatic stellate cell; TGF-β, transforming growth factor β; Smad, A family of proteins that mediate the signaling of TGF-β family ligands; TNF-α, tumor necrosis factor alpha; MYC, V-myc Avian Myelocytomatosis Viral Oncogene Homolog; STAT3, signal transducer and activator of transcription 3; CAPRIN1, cell cycle-related antiproliferative intestinal protein 1; SPOP, speckle type POZ protein; YAP, Yes 1 associated transcriptional regulator; TAZ, transcriptional co-activator with PDZ-binding motif.

LLPS has emerged as a fundamental mechanism organizing biomolecules into dynamic, membraneless compartments within cells. While its role in neurodegeneration has been increasingly explored, recent studies have begun to uncover its involvement in CLDs. As shown in Figure 4, the molecular events mediated by LLPS progressively impact the stages from a healthy liver to NAFLD, fibrosis, and HCC, with significant changes in cellular signaling, metabolism, and gene regulation.

### Liquid-liquid phase separation in non-alcoholic fatty liver disease

#### Liquid-liquid phase separation-related dynamics in lipid metabolic imbalance

NAFLD represents the most prevalent chronic liver disorder worldwide. Abnormal lipid metabolism is a core mechanism in the pathogenesis of NAFLD.^127^ Metabolic disorders, particularly IR, are key drivers of chronic inflammation and alterations in lipid metabolism.^128^ IR leads to increased hepatic lipid synthesis and impaired lipid clearance, resulting in hepatic lipid deposition and lipotoxicity.^129^ Studies have shown that LLPS participates in the dynamic organization of lipid metabolism-related proteins and, under certain conditions, contributes to abnormal lipid deposition.^130^ For example, excess lipids and perilipin family proteins such as PLIN1, which contain intrinsically disordered regions, can undergo LLPS through multivalent interactions, leading to the formation of abnormal lipid droplets. These condensates promote the nucleation and growth of triglyceride cores by spatially isolating them, while also limiting access to lipolytic enzymes such as ATGL. As a result, lipid droplets become enlarged, contributing to steatosis.^131^^,^^132^ Additionally, LLPS regulates metabolic pathways by altering the subcellular localization and activity of metabolic enzymes and cofactors. For instance, enzymes involved in lipid metabolism, such as fatty acid synthase (FASN) and acetyl-CoA carboxylase (ACC), may aggregate via LLPS to form localized metabolic hubs, thereby modulating lipid synthesis and breakdown.^133^ When this balance is disrupted, excessive fatty acids accumulate in hepatocytes, resulting in steatosis. In patients with NAFLD, LLPS involving 17-β-hydroxysteroid dehydrogenase 13 (HSD17B13) occurs around hepatic lipid droplets, enhancing its enzymatic function and increasing the biosynthesis of platelet-activating factor (PAF).^134^ This promotes fibrinogen synthesis and leukocyte adhesion, ultimately triggering hepatic inflammation.^135^ Furthermore, high-fat diets can activate the cGAS-STING signaling pathway in adipose tissue, which not only induces IR but also causes metabolic dysfunction.^136^ In hepatocytes, lipid overload triggers replication stress and DNA damage, further upregulating cGAS-STING signaling.^137^ These findings underscore the intricate connection between inflammatory responses and metabolic dysfunction. In summary, LLPS mediates the dynamic assembly of key metabolic enzymes and regulatory factors, coordinating the complex network of intracellular metabolism and inflammatory responses, thereby influencing hepatocyte homeostasis and pathological progression. The interplay between lipid accumulation, inflammatory signaling, and DNA damage accelerates the development of liver fibrosis and hepatocellular carcinoma. Targeting LLPS mechanisms may disrupt the vicious cycle between metabolic imbalance and chronic inflammation, offering a novel therapeutic avenue for NAFLD and its related complications.

#### Liquid-liquid phase separation amplifies hepatic inflammatory signaling

During the progression of NAFLD, chronic inflammation represents a key pathological driver of the transition to NASH, which markedly increases the risk of liver cirrhosis and HCC.^138^ As a fundamental mechanism of biomolecular organization, LLPS is deeply involved in the transmission and amplification of hepatic inflammatory signaling. During the NAFLD/NASH stages, hepatocytes are exposed to multiple stressors, including lipotoxicity, oxidative stress, and ER stress.^139^ These stress signals continuously activate the IKK-NF-κB signaling pathway through various pattern recognition receptors and kinases.^140^ Due to the amplification properties of LLPS, even low-intensity stimuli can elicit a robust NF-κB response, leading to the excessive production of inflammatory cytokines by hepatocytes.^141^ This localized “cytokine storm” not only directly induces hepatocyte apoptosis and injury but also creates a pro-inflammatory hepatic microenvironment that further exacerbates disease progression. For instance, in hepatocytes, the binding of TNF-α to its receptor induces signaling proteins such as TRADD and RIPK1 to undergo LLPS, forming signaling complexes that activate inflammation-related transcription factors such as NF-κB.^142^ This activation promotes the expression of inflammatory genes, exacerbates inflammatory responses, and drives NAFLD progression. Moreover, in patients with NAFLD, NF-κB expression is markedly elevated and has been shown to correlate positively with the degree of liver injury.^143^ This upregulation is thought to contribute to hepatic insulin resistance and suggests that NF-κB may also play a critical role in promoting obesity-associated HCC, potentially through the regulation of IL-6 and TNF signaling.^144^ In parallel, inflammation and various stress responses triggered by lipid metabolic dysregulation in hepatocytes may promote the LLPS-mediated assembly of upstream regulators in the Hippo signaling pathway. These condensates can influence the nuclear localization and transcriptional activity of YAP and TAZ, further exacerbating hepatic steatosis and inflammatory responses.^145^

Notably, LLPS also plays a crucial role in inflammasome activation and immune responses. LLPS facilitates the aggregation of inflammasome components, which act as signal amplifiers upon formation within cells, activating key pro-inflammatory cytokines such as IL-1β and IL-18. These cytokines contribute to the inflammatory response in NASH. For example, the activation of IRE1α promotes the activation of the NLRP3 inflammasome, intensifying inflammation.^146^ Studies in mice lacking inflammasome components have shown reduced hepatic triglyceride content, smaller adipocyte size, and decreased macrophage infiltration in adipose tissue,^147^ suggesting that targeting inflammasomes could be a potential therapeutic strategy for NAFLD. Furthermore, Wu et al. discovered that dsRNA interacts with NLRP6, inducing its LLPS and subsequently activating inflammasomes to promote antimicrobial immune responses in the gut and liver.^148^ This finding highlights the significant role of LLPS in inflammasome formation and antiviral immunity. In addition, metabolic and oxidative stress-induced mitochondrial injury and nuclear DNA damage in hepatocytes can lead to the cytosolic release of large amounts of mitochondrial and nuclear DNA. These endogenous DNA fragments act as damage-associated molecular patterns (DAMPs), which are recognized by cGAS and activate the cGAS-STING pathway through LLPS.^149^ This persistent, low-grade inflammatory signaling is considered a key contributor to the transition from NAFLD to NASH and liver fibrosis. Wang et al. reported that STING expression is significantly elevated in the livers of patients with NASH, increasing with the severity of inflammation and fibrosis.^150^ Luo et al. demonstrated that the detrimental effects of STING in NASH are mediated through enhanced pro-inflammatory responses in macrophages.^151^ Under lipid overload, STING acts as a mitochondrial DNA sensor in hepatic Kupffer cells, inducing NF-κB-dependent inflammation in NASH.^152^ LLPS has been shown to enhance both the initiation and persistence of inflammatory signaling by regulating multiple pathways, including NF-κB activation, inflammasome assembly, and the cGAS-STING axis. Through these mechanisms, LLPS plays a central role in driving the pathological transition from NAFLD to NASH. Targeting LLPS to restore inflammatory homeostasis may offer a promising therapeutic strategy to slow disease progression and improve clinical outcomes in patients with NAFLD.

#### Liquid-liquid phase separation regulates endoplasmic reticulum stress and unfolded protein response

The ER plays a critical role in the folding of secretory and transmembrane proteins, calcium homeostasis, and lipid biosynthesis.^153^ However, when a large number of unfolded or misfolded proteins accumulate in the ER, cells initiate the unfolded protein response (UPR).^154^ Certain UPR-related signaling proteins and molecular chaperones form condensates via LLPS, regulating processes such as protein folding and cell survival. In the liver tissues of patients with NAFLD, lipid stress, such as lipid overload and impaired VLDL-TG assembly, leads to ER morphological abnormalities and functional disruption, triggering ER stress responses.^155^ The activation of IRE1α, a transmembrane protein kinase, is a pivotal step in the UPR.^156^ The accumulation of unfolded proteins in the ER promotes the dimerization of IRE1α on the ER membrane, activating its RNase activity through autophosphorylation.^157^ The RNase activity of IRE1α splices XBP-1 mRNA, producing the active transcription factor XBP-1s, which regulates the expression of genes involved in lipid metabolism and cell survival.^158^ This UPR activation, mediated by LLPS, plays a critical role in the progression of NAFLD, as persistent UPR activation can lead to cellular dysfunction, such as abnormal lipid metabolism, exacerbating the disease. Furthermore, IRE1α dynamically colocalizes with stress granules (SGs) formed via LLPS through its cytoplasmic intrinsically disordered region.^159^ This colocalization is essential for IRE1α function, as it facilitates signal transduction and mRNA splicing, thereby influencing gene expression. IRE1α also activates the NF-κB pathway through its kinase activity, enabling NF-κB transcription factors to translocate into the nucleus and activate the expression of inflammation-related genes. NF-κB plays multifaceted roles in NASH, acting as a central factor in liver injury, fibrosis, and even HCC. It also drives a vicious cycle of damage and inflammation by promoting the release of mediators such as IL-1β and TNFα from hepatic Kupffer cells. This LLPS-mediated signal transduction is crucial for cellular responses to ER stress.^160^ In NAFLD, dysregulated protein folding can lead to the accumulation of misfolded proteins, such as cytokeratin 18. Under these conditions, the autophagy receptor p62 recognizes and sequesters misfolded proteins to form Mallory-Denk bodies (MDBs), a type of intracellular protein aggregate commonly observed in hepatocytes.^161^ Through LLPS, p62 mediates the condensation and compartmentalization of these aberrant proteins into droplet-like structures, thereby preventing their uncontrolled diffusion and protecting cells from associated cytotoxic stress.^162^ This mechanism plays a protective role in mitigating hepatic inflammatory responses. However, under prolonged stress conditions, excessive accumulation and persistent aggregation of p62 may contribute to extracellular matrix deposition and exacerbate hepatocellular injury, ultimately promoting inflammation and fibrogenesis.^163^ Therefore, LLPS plays a vital role in ER stress by participating in the formation of stress granules, modulating signal transduction, and regulating gene expression. These processes collectively influence the progression of NAFLD. The study of LLPS offers new perspectives for understanding the pathological mechanisms of NAFLD and may provide novel therapeutic targets for its treatment.

### Liquid-liquid phase separation in liver fibrosis

#### Liquid-liquid phase separation-induced extracellular matrix pathological remodeling in liver fibrosis

There is a close relationship between liver fibrosis and NAFLD, with the latter being a common cause of the former. The natural progression of NAFLD typically begins with hepatic fat accumulation, which, if uncontrolled, may develop into NASH and subsequently lead to liver fibrosis.^127^ Liver fibrosis is a reparative response to chronic injury characterized by the excessive deposition of ECM, disrupting normal liver structure and function. This progressive fibrosis can lead to cirrhosis, portal hypertension, and liver failure, making it a leading cause of liver-related mortality in patients with NAFLD.^164^ During liver fibrosis, the synthesis of ECM components, such as collagen and fibronectin, increases significantly. Some ECM-related proteins possess IDRs, which enable them to form aggregates through LLPS.^52^ For example, collagen molecules can undergo LLPS under specific conditions, transitioning into highly ordered fibrillar aggregates.^165^ These fibrillar structures are more resistant to degradation than their normal counterparts, resulting in their accumulation in liver tissue. As the quantity of these aggregates increases, parenchymal cells in the liver become progressively isolated by the expanding ECM, disrupting the original architecture of the liver and perpetuating the progression of fibrosis.

In addition to classical biochemical signaling pathways, emerging research has revealed that HSCs may also sense mechanical tension from the collagen matrix through LLPS-mediated mechanisms. Surface receptors on HSCs that interact with collagen can engage in multivalent interactions, driving LLPS and leading to the formation of biomolecular condensates that function as mechanosensory hubs. These condensates integrate physical tension cues from the extracellular matrix and coordinate HSCs’ directional migration as well as the formation of characteristic fibrous septa.^166^ This discovery provides new insight into how mechanical signals may regulate structural remodeling during liver fibrosis via LLPS, thereby expanding our understanding of the regulatory network governing HSCs activation.

#### Liquid-liquid phase separation mediates the functional transition of HSCs in liver fibrosis

The activation of HSCs is a pivotal step in the process of liver fibrosis.^167^ Upon hepatic injury, quiescent HSCs undergo activation and transdifferentiate into myofibroblast-like cells.^168^ This phenotypic transition is characterized by enhanced proliferative capacity, increased contractility, and robust production of ECM components.^169^ The excessive accumulation of ECM within the liver parenchyma ultimately contributes to the development and progression of liver fibrosis.^170^ Emerging evidence suggests that LLPS is deeply involved in the signal transduction pathways and molecular regulatory networks associated with HSC activation.^171^ Biomolecular condensates formed via LLPS may serve as concentrated platforms for signaling molecules, thereby facilitating the activation of key fibrogenic pathways such as TGF-β/Smad, NF-κB, and MAPK. Through this mechanism, LLPS contributes to the spatial and temporal organization of signaling events that drive the fibrotic response.^172^^,^^173^ For instance, upon activation, the NF-κB pathway translocates to the nucleus, where it induces the transcription of pro-inflammatory and pro-fibrotic cytokines such as TNFα, IL-1β, CCL5, and TGFβ.^174^ These cytokines not only directly contribute to the activation and persistence of HSCs, but also promote lipid accumulation and apoptosis in hepatocytes, thereby establishing a vicious cycle that exacerbates liver inflammation and fibrogenesis.^175^ Among these factors, TGF-β1 plays a crucial role in fibrosis progression by promoting collagen synthesis and inhibiting its degradation.^176^ Upon binding to its receptor, TGF-β activates Smad2 and Smad3 (R-Smads), which then form complexes with Smad4 (Co-Smad) and translocate to the nucleus to activate fibrosis-related genes. Recent studies have shown that RNA-binding proteins such as SFPQ can drive LLPS through their prion-like domains (PrLDs), thereby sequestering Smad4 and disrupting TGF-β tumor-suppressive signaling, suggesting that LLPS may play a significant regulatory role in the TGF-β/Smad pathway.^177^

Furthermore, LLPS enhances the transcriptional activity of fibrotic genes by promoting the condensation of signaling complexes. In the context of NASH, YAP/TAZ, key components of the Hippo signaling pathway, are closely associated with the progression of liver fibrosis.^178^ The activation of YAP/TAZ regulates myofibroblast activation through multiple mechanisms, accelerating the fibrotic process. Wang et al. found that the TAZ protein in hepatocytes interacts with TEAD family transcription factors to activate the transcription of the Indian hedgehog (Ihh) gene. Ihh, a secreted factor, activates fibrogenic genes in HSCs, thereby promoting fibrosis. By increasing Ihh secretion, TAZ activation in hepatocytes upregulates the expression of pro-fibrotic genes in HSCs, driving the progression of liver fibrosis.^179^ In addition to producing excessive ECM, activated HSCs continuously release pro-inflammatory mediators that sustain hepatic inflammation. This persistent inflammatory state further reinforces the activation of HSCs, establishing a self-perpetuating “inflammation-fibrosis” loop. Over time, this vicious cycle may lead to progressive liver damage and, ultimately, organ failure.^180^

Notably, recent studies have revealed that excessive physical exercise can lead to elevated lactate levels, which promote the lactylation of SORBS3 in skeletal muscle. This modification drives LLPS and leads to the formation of lactate-enriched biomolecular condensates—referred to as “lactasomes”—within small extracellular vesicles (sEVs) enriched in FBXO2. These vesicles are transported via the bloodstream to the liver, where they induce hepatocyte apoptosis and indirectly activate HSCs.^181^ Interestingly, salidroside, a bioactive compound derived from *Rhodiola*, has been shown to effectively inhibit this pathological process.^181^ In addition, diallyl trisulfide (DATS), a natural compound derived from garlic, has been reported to induce LLPS of the RAB18 protein, thereby establishing a positive feedback loop that promotes the formation of mitochondria-associated membranes and inhibits lipophagy. This process ultimately triggers selective cuproptosis in activated HSCs, offering a novel therapeutic strategy for targeted clearance of fibrogenic cells.^182^

#### Liquid-liquid phase separation in the inflammation-fibrosis loop during liver fibrogenesis

Liver fibrosis is a chronic condition resulting from sustained hepatic injury and is typically accompanied by immune-inflammatory responses within the liver.^183^ Recent studies have underscored the essential role of LLPS in regulating immune cell functions, the release of inflammatory mediators, and intercellular signaling.^184^ In the context of liver fibrosis, LLPS appears to modulate the crosstalk between immune cells——particularly Kupffer cells (KCs) and HSCs, thereby influencing the inflammatory environment and contributing to fibrotic progression.^184^ KCs, the liver-resident macrophages, act as the first line of defense during liver injury.^185^ Upon sensing tissue damage or pathogenic stimuli, KCs secrete pro-inflammatory cytokines such as IL-1β and TNFα, initiating downstream immune responses. Within KCs, LLPS mediates the formation and activation of inflammasomes, a process that facilitates the maturation and release of these cytokines.^184^ In turn, IL-1β and TNFα promote the activation of HSCs, enhancing collagen synthesis and extracellular matrix deposition, thereby amplifying hepatic inflammation and accelerating fibrotic progression.^186^ Moreover, macrophage-derived IL-1β and TNFα directly promote NF-κB activation in HSCs, supporting their survival.^187^ The depletion of macrophages reduces NF-κB activation in HSCs, underscoring the pivotal role of macrophages in HSC activation.^186^

LLPS within KCs may also influence their chemotactic effect on HSCs. By regulating the condensation and release of chemokines, LLPS enhances the local concentration of these signaling molecules, effectively attracting HSCs to sites of injury and promoting their activation.^188^ Furthermore, condensates formed via LLPS in KCs might directly interact with specific receptors on the HSC membrane. This interaction represents a novel form of intercellular communication, transmitting activation signals and activating intracellular pathways such as the Akt/mTOR pathway, which plays an essential role in the onset and progression of liver fibrosis. LLPS also affects the metabolic state of KCs, providing the energy and biomolecules necessary for inflammatory responses. Additionally, LLPS may influence the recruitment and homing of immune cells within the liver, attracting more immune cells to infiltrate hepatic tissue and exacerbating inflammation and fibrosis severity. In summary, these mechanisms collectively highlight the critical role of LLPS in liver fibrosis, providing new insights into therapeutic strategies targeting LLPS to disrupt the inflammation-fibrosis loop.

### Liquid-liquid phase separation in liver cancer

#### Liquid-liquid phase separation in gene fusion and transcriptional dysregulation

Gene fusion events play a pivotal role in the development of liver cancer, with LLPS emerging as a critical influencing factor. Chromosomal translocations can produce novel fusion genes whose products may undergo aberrant phase separation.^189^ The IDRs of fusion proteins can drive the formation of functionally specialized condensates through LLPS, which disrupt normal cellular signaling pathways and promote the proliferation and survival of cancer cells.^190^ This aberrant phase separation phenomenon is not restricted to fusion genes; other transcription factors and proteins can also promote tumorigenesis and progression through similar LLPS-dependent mechanisms. For instance, FOXM1 is capable of undergoing LLPS with FKH-shared DNA elements, resulting in the formation of abnormal condensates that spatially segregate transcriptional machinery within the nucleus and enhance the transcriptional efficiency of specific target genes, thereby facilitating tumor growth and metastasis.^191^ The dysregulated phase separation of FOXM1 may manifest as an increase in condensate number and size, enhanced stability, or altered subnuclear localization—all of which contribute to the excessive activation of its oncogenic target genes, ultimately accelerating cell proliferation, angiogenesis, and metastatic dissemination.^191^ Recent studies have revealed that ASPM, a protein highly expressed in HCC, interacts with FOXM1 via its IDRs to drive LLPS, forming intranuclear biomolecular condensates. These condensates effectively sequester FOXM1 within the nucleus, markedly increasing its protein stability and substantially enhancing its transcriptional activation of downstream cell cycle- and proliferation-related oncogenes, such as CCND1. This interaction establishes a self-reinforcing positive feedback loop that drives the malignant progression of HCC.^192^

Meanwhile, the functional significance of long non-coding RNAs (lncRNAs) in HCC is increasingly being recognized. Through interactions with RNA-binding proteins (RBPs), lncRNAs modulate RBP activity and participate in key cellular processes. Importantly, by engaging in LLPS, lncRNAs play critical roles in DNA damage repair, tissue homeostasis, and tumor progression.^193^ For instance, lncRNA-MALAT1 is upregulated in liver cancer tissues and interacts with multiple RBPs via LLPS to form ribonucleoprotein (RNP) granules, regulating gene expression and promoting invasion and metastasis of cancer cells.^194^ Similarly, lncRNA-ZNF32-AS2 engages in LLPS and facilitates the proliferation and migration of liver cancer cells.^195^ Recent studies have identified a novel lncRNA-NEAT1, within extracellular vesicles (EVs) derived from cancer-associated fibroblasts (CAFs), as reported by Chen et al. NEAT1 is markedly overexpressed in HCC and is associated with poor clinical prognosis. Mechanistically, NEAT1 directly interacts with the YAP protein, facilitating its LLPS, thereby promoting HCC proliferation, metastasis, and the self-renewal capacity of liver cancer stem cells (CSCs).^196^ Circular RNAs (circRNAs) also influence liver cancer progression through LLPS. For example, circASH2 promotes nuclear LLPS of YBX1 and assembles complexes with hnRNPs to target TPM4 transcripts, interfering with their splicing and accelerating their degradation, effectively suppressing the invasion and metastasis of liver cancer.^197^

Aberrant condensates formed through LLPS have been increasingly recognized as a mechanism by which transcription factor dysregulation may be involved in malignant progression. These condensates bind to the promoter regions of numerous target genes, altering gene expression patterns and disrupting normal transcriptional programs.^198^ For example, the transcription factor MYC is abnormally activated in liver cancer cells. Its associated zinc finger protein, MAZ, which contains IDRs, interacts with G4 sequences and forms phase-separated condensates via LLPS, concentrating in specific nuclear regions to activate the expression of proliferative genes such as CCND1, thereby accelerating liver cancer cell proliferation.^199^^,^^200^

Moreover, the p53 protein, a pivotal tumor suppressor, plays a central role in maintaining genomic stability and preventing malignant transformation. Recent evidence suggests that p53 can form dynamic, functional condensates via LLPS, contributing to DNA repair and transcriptional regulation under physiological conditions.^201^^,^^202^ However, under certain pathological contexts—such as gene mutations, cellular stress, or dysregulated post-translational modifications—these metastable p53 droplets may undergo a liquid-to-solid phase transition, resulting in the formation of dysfunctional solid aggregates with potential oncogenic properties.^25^^,^^203^ On one hand, the rigid architecture of these aggregates may impair p53’s ability to bind target DNA sequences, thereby compromising its tumor-suppressive functions.^204^ On the other hand, mutant p53 proteins have been shown to exhibit prion-like behavior, acting as pathological seeds that recruit wild-type p53 and its homologs, p63 and p73, into amyloid-like co-aggregates.^205^^,^^206^ This aberrant phase transition may lead to dominant-negative effects, whereby not only is the function of mutant p53 lost, but the tumor-suppressive activities of its family members are also inhibited.^204^ In HCC, TP53 is the second most frequently mutated gene, with alterations found in over 30% of cases. Among these, the R249S hotspot mutation—commonly observed in HCC—has been reported to markedly enhance the aggregation propensity of p53, accelerating the liquid-to-solid transition and promoting the formation of solid aggregates with amyloid-like features.^203^ These aggregates have been implicated in promoting liver cancer progression.^203^ Collectively, these findings suggest that targeting the pathological aggregation of p53 may represent a potential therapeutic strategy to restore its physiological function in HCC.^202^^,^^203^

In addition to genetic mutations, p53 function can also be modulated through its dynamic subcellular localization and interactions with non-coding RNAs. Yuan et al. demonstrated that the oncogenic lncRNA-TLNC1 binds to TPR, inducing nuclear-to-cytoplasmic translocation of p53, thereby inhibiting its transcriptional activity on target genes.^207^ This LLPS-mediated p53 relocalization weakens its DNA repair and tumor-suppressive functions, significantly advancing HCC progression. Similarly, lncRNA MEG3 has been shown to modulate the expression of p53 target genes in hepatocytes, thereby influencing cell fate decisions.^208^ These findings highlight the multifaceted regulation of p53 at both the genetic and phase separation levels, which plays a key role in liver tumorigenesis.

While p53 represents a classical tumor suppressor whose function is often compromised through LLPS-mediated dysregulation, other transcription factors with oncogenic potential are similarly regulated via phase separation. Signal transducer and activator of transcription 3 (STAT3) is another key player in signal transduction and transcriptional activation.^209^ In liver cancer cells, growth factors and cytokines can activate STAT3, which contains LLPS-prone domains, including the N-terminal domain (NTD), coiled-coil domain (CCD), and SRC homology 2 (SH2) domain.^210^ Upon external stimulation, STAT3 may form condensates via LLPS, activating genes associated with proliferation, angiogenesis, and immune evasion in liver cancer. For example, STAT3 upregulates the expression of vascular endothelial growth factor (VEGF), promoting tumor angiogenesis and ensuring an adequate supply of nutrients and oxygen for liver cancer cells.^211^ Additionally, STAT3 modulates the expression of immune checkpoint molecules, enabling liver cancer cells to evade immune surveillance, thereby facilitating tumor progression.^212^

#### Liquid-liquid phase separation in aberrant protein aggregation and functional dysregulation

In the complex mechanisms underlying HCC development, many tumor-associated proteins form aggregates through LLPS, altering intracellular signaling environments and intertwining with various molecular pathways. On one hand, Chen et al. demonstrated that the circRNA circVAMP3 interacts with the CAPRIN1 protein to induce its phase separation, promoting SG formation and inhibiting c-Myc translation and MYC proto-oncogene protein expression.^213^ This highlights the role of LLPS in regulating HCC-related gene expression at the interaction level of non-coding RNAs and proteins. On the other hand, genetic mutations or environmental factors may alter protein structure and function, resulting in aberrant phase separation, which continuously activates downstream signaling pathways, promoting HCC progression. The E3 ubiquitin ligase SPOP, a substrate adaptor of the cullin3-RING ubiquitin ligase, localizes to nuclear speckles and suppresses tumorigenesis by targeting key oncogenic proteins for degradation.^214^ The oligomerization and LLPS of SPOP are crucial for its function, and cancer-associated SPOP mutations can disrupt these processes, impairing the function of wild-type SPOP.^215^ For example, cancer-associated SPOP mutants, such as S119N, disrupt interactions with novel substrates such as cAMP response element-binding protein 5 (CREB5), impairing CREB5 ubiquitination and leading to receptor tyrosine kinase MET pathway activation, which enhances the metastatic potential of HCC cells.^216^ Similarly, He et al. reported that the E3 ubiquitin ligase RNF214 is highly expressed in HCC and acts as an oncogene, promoting HCC proliferation, migration, and metastasis.^217^ Its coiled-coil (CC) domain mediates phase separation. Recent studies have found that RNF214 enhances the transcriptional activity of the YAP-TEAD complex, further emphasizing the role of LLPS in HCC progression.^218^ Recent studies have uncovered a novel mechanism by which the atypical kinase RIOK1 drives tumorigenesis through LLPS. RIOK1 is markedly overexpressed in HCC and promotes the formation of SGs via LLPS. These SGs specifically sequester PTEN mRNA, thereby reducing its translation and relieving the inhibition of oncogenic signaling pathways such as PI3K-AKT-mTOR, ultimately promoting tumor growth.^219^ In addition, the PKA fusion oncoprotein DnaJB1-PKAcat, associated with fibrolamellar carcinoma (FLC), undergoes aberrant LLPS through its prion-like domain, hijacking the regulatory subunit Riα and preventing its degradation. This disruption leads to dysregulated cAMP signaling and cellular transformation, further highlighting that aberrant phase separation may act as a driving force in tumor initiation.^220^

Additionally, the Hippo signaling pathway negatively regulates nuclear YAP and TAZ condensate formation via LATS-mediated phosphorylation.^221^ As transcriptional co-activators, YAP and TAZ play a critical role in driving multiple malignant phenotypes of HCC cells through the dysregulation of their protein functions.^222^ In HCC, YAP and TAZ are frequently overexpressed and exhibit widespread nuclear accumulation-alterations that can enhance the proliferative, invasive, and migratory capacities of cancer cells, thereby accelerating disease progression.^223^ Lu et al. demonstrated that the deletion of Mst1 and Mst2 in mouse hepatocytes leads to the dysregulation of LATS phosphorylation of YAP/TAZ, resulting in YAP activation. This activation caused significant liver enlargement and multiple tumor nodules within 5-6 months, highlighting YAP’s potent role in driving hepatocyte proliferation and tumor formation.^224^ In addition, YAP protein has been shown to facilitate epithelial-mesenchymal transition (EMT) in HCC cells by orchestrating key signaling cascades within the tumor microenvironment, notably the TGF-β/Smad pathway, thereby augmenting the metastatic dissemination and invasive capabilities of malignant cells.^225^ This process involves the interaction of the YAP protein with other pathway-related proteins and the regulation of downstream gene expression. These functions are fundamentally dependent on the intrinsic activity and interaction properties of YAP protein. YAP and TAZ condensates also support cell proliferation and resistance to anti-PD-1 immunotherapy by modulating the expression of genes associated with drug metabolism and efflux, influencing the therapeutic response in HCC.^226^ Beyond upstream regulatory factors, a lipid-associated lncRNA, small nucleolar RNA host gene 9 (SNHG9), has been identified as an oncogenic lncRNA that promotes tumor progression. SNHG9 drives the formation of aberrant LATS1 condensates and suppresses Hippo pathway activity, thereby contributing to the regulation of cell proliferation and differentiation processes.^79^

Furthermore, the dysregulation of protein quality control mechanisms is associated with LLPS. In normal cells, molecular chaperones and the proteasome system prevent abnormal protein aggregation.^227^ However, in HCC cells, these systems may be impaired, leading to misfolded proteins aggregating through LLPS and disrupting normal cellular functions.^228^ Heat shock proteins (HSPs) are essential molecular chaperones that maintain proteostasis within cells.^229^ In HCC cells, HSP function may be compromised, leading to the improper folding and mislocalization of oncogenic proteins such as RAS. This results in aberrant protein aggregation and sustained activation of downstream signaling pathways, ultimately promoting uncontrolled proliferation and angiogenesis in cancer cells.^230^

#### Liquid-liquid phase separation in metabolic dysregulation and microenvironmental factors

The metabolism of HCC cells undergoes significant alterations. By impacting metabolic pathways in the tumor microenvironment, LLPS provides energy and essential biomolecules necessary for tumor cell growth and invasion.^231^ IR is a critical factor in the progression of NAFLD to HCC, reducing hepatic insulin sensitivity and impairing glycogen synthesis and degradation.^232^ In some cases, metabolic abnormalities lead to excessive glycogen accumulation in the liver. Lin et al. demonstrated that glycogen accumulation represents a key oncogenic event in liver malignancy initiation. Accumulated glycogen undergoes spontaneous LLPS, facilitating the assembly of the Laforin-Mst1/2 complex and sequestering Hippo kinases Mst1/2 in glycogen droplets. This inactivates the tumor-suppressive Hippo signaling pathway and activates the downstream oncogene YAP, driving tumor initiation.^233^ This finding marks the first discovery that a macromolecular metabolite such as glycogen can regulate tissue homeostasis through LLPS. Recent studies further reveal that the metabolic enzyme glycogen synthase 1 (GYS1), a key regulator of glycogenesis, undergoes LLPS with the nuclear protein NONO/p54nrb. This GYS1-NONO condensate dynamically regulates nuclear glycogen synthesis and spatiotemporal control of myogenic differentiation.^234^ In the context of liver cancer, aberrant GYS1 phase separation may disrupt glycogen homeostasis, promoting metabolic reprogramming and enhancing tumor cell proliferation. Furthermore, NONO’s role in RNA processing suggests that GYS1 condensates could couple glycogen metabolism with transcriptional regulation, providing a novel mechanism linking energy storage to oncogenic signaling.

Additionally, extracellular microenvironmental factors such as hypoxia and inflammatory cytokines can induce intracellular LLPS processes.^235^ In HCC tissues, cells often experience hypoxia due to insufficient vascular supply.^236^ Under hypoxic conditions, HCC cells form non-membranous particles called “G-bodies,” which aggregate glycolytic enzymes through LLPS. This aggregation accelerates glucose conversion to lactate, providing energy and biosynthetic precursors for cancer cells in the hypoxic microenvironment.^237^ Thus, G-bodies and their associated LLPS mechanisms likely play critical roles in HCC cells’ adaptation to hypoxia, maintenance of metabolic homeostasis, and tumor progression. Recent studies have revealed that hypoxic microenvironments can induce LLPS of the transcription factor ZHX2 via its IDRs enriched in proline residues, thereby reshaping chromatin architecture and promoting tumor metastasis.^235^ In addition, Hypoxia also upregulates hypoxia-inducible factors (HIFs), which interact with other transcription factors via LLPS to regulate gene expression associated with angiogenesis and metabolic adaptation, creating favorable conditions for HCC cell growth.^238^ For example, hypoxia-induced HIF-1α interacts with the p300 transcription factor through LLPS, forming condensates that bind to the promoter region of the VEGF gene and upregulate its expression.^239^ VEGF promotes tumor angiogenesis, improving blood supply to HCC cells and thereby facilitating tumor growth and metastasis.^240^ LLPS may contribute to abnormal lipid deposition and is implicated in exacerbating hepatic steatosis under metabolic stress. In conclusion, understanding the mechanisms by which LLPS influences HCC cell behavior is crucial for developing novel therapeutic NF-κB condensates and sustaining cytokine expression strategies targeting these processes, which may improve the prognosis of patients with HCC. The identification of LLPS-related biomarkers could also enhance prognosis evaluation and inform treatment decisions for liver cancer.

In chronic liver diseases, LLPS regulates the aggregation and function of various molecules, thereby influencing key pathological processes such as lipid accumulation, inflammation, and fibrosis. It also plays a critical role in cellular dysfunction, tumor progression, and immune evasion. As illustrated in Table 3, the relationship between these molecules and LLPS, along with their pathological roles in chronic liver disease, is clearly demonstrated. These insights offer a new perspective for developing therapeutic strategies targeting chronic liver disorders.

**Table 3:** LLPS-related molecules and their functions in chronic liver disease

Condensates	Connection with LLPS	Pathological Function	Reference
cGAS-STING	Recognizes mitochondrial DNA, activates inflammatory pathways via LLPS	Promotes Kupffer cell release of IL-1β, driving fibrosis	Cho and Gupta^149^; Wang et al.^150^; Yu et al.^152^
NF-κB-NEMO	Multivalent ubiquitin chains drive LLPS, activating the IKK complex	Induces hepatocyte apoptosis, activates HSCs, promoting fibrotic gene expression	Mobeen et al.^143^; Kern et al.^144^
YAP/TAZ	Forms nuclear condensates with TEAD4, regulating super-enhancers	Promotes HSCs activation and EMT, enhances tumor invasiveness	Kiang et al.^145^
p62	Aggregation of ubiquitinated proteins via the UBA domain, forming Mallory-Denk bodies	Autophagy dysfunction leads to chronic inflammation, promotes fibrosis-cancer transition	Ke^161^; Huang et al.^162^
SPOP	Mutant disrupts phase separation, leading to CREB5/MET pathway activation	Enhances tumor metastasis, inhibits wild-type SPOP tumor suppressor function	Gong et al.^216^
PERILIPIN family proteins	LLPS mediates lipid droplet fusion, forming large lipid droplets	Lipotoxicity-induced ER stress, triggers UPR and inflammation	Chandrasekaran et al.^131^
TGF-β/Smad	SFPQ phase separation isolates Smad4, inhibiting tumor suppressor signaling	Promotes HSCs collagen synthesis, inhibits ECM degradation	Xiao et al.^177^
STAT3	SH2 domain-driven phase separation, forming transcriptionally active condensates	Upregulates VEGF, promotes angiogenesis, evades immune surveillance	Xu et al.^211^
HSD17B13	Phase separation around lipid droplets enhances PAF synthesis	Promotes platelet activation, aggravates sinusoidal microthrombosis	Ye et al.^134^
FUS/TLS	DNA damage site phase separation, recruits the PARP1 repair complex	Genomic instability promotes tumor mutation accumulation	Singatulina et al.^120^

## Conclusion and future perspectives

LLPS, as a dynamic and finely regulated biomolecular organization mechanism, is gradually revealing its core role in cell fate determination and disease progression.^184^ Especially in the field of chronic liver diseases, increasing evidence suggests that LLPS plays a key role in the progression from NAFLD to liver fibrosis and even HCC by regulating lipid metabolism, inflammatory signaling, endoplasmic reticulum stress, and fibrotic responses.^184^ Its dysregulation often leads to lipid metabolism disorders, amplified inflammatory signaling, activation of HSCs, and abnormal gene transcription, thereby driving irreversible damage to liver structure and function.^134^^,^^241^

In the early stages of NAFLD, LLPS orchestrates the dynamic assembly of lipid droplet-associated proteins and metabolic enzymes, thereby maintaining lipid metabolic homeostasis.^130^ When LLPS is disrupted, lipid accumulation exacerbates ER stress and oxidative stress, triggering inflammatory responses and driving disease progression.^242^ Meanwhile, LLPS amplifies inflammatory signaling and sustains a chronic inflammatory microenvironment by organizing key components of the NF-κB signaling complex, inflammasomes, and the cGAS-STING pathway. This persistent inflammation leads to hepatocellular injury and continuous activation of HSCs, thereby exacerbating fibrosis. Moreover, in HCC, aberrant LLPS driven by gene fusions, transcriptional dysregulation, protein aggregation, and metabolic reprogramming leads to the formation of pathological condensates that disrupt intracellular signaling pathways, activate oncogenes, impair tumor suppressor functions, and remodel the tumor microenvironment. In addition, LLPS mediates dynamic interactions among oncoproteins, non-coding RNAs, and stress-responsive elements, thereby promoting tumor cell proliferation, metastasis, immune evasion, and therapeutic resistance.

Despite emerging evidence highlighting the potential role of LLPS in chronic liver diseases, numerous critical questions remain unanswered. These include the specific roles of LLPS across different stages of disease progression, its crosstalk with classical inflammatory, metabolic, and cell death signaling pathways, whether LLPS-regulated molecular mechanisms exhibit disease specificity, and how LLPS influences intercellular interactions within the complex hepatic microenvironment to drive pathogenesis. Addressing these questions is not only essential for elucidating the molecular underpinnings of chronic liver diseases but also holds significant promise for the development of novel therapeutic strategies.

Therapeutically, LLPS presents a new avenue for targeted intervention in chronic liver disorders. Recent studies have demonstrated that the small molecule RQ can induce the condensation of β-catenin into liquid-like droplets through LLPS, thereby preventing its nuclear translocation and subsequent activation of oncogenic transcriptional programs. This mechanism has been shown to significantly suppress hepatocellular carcinoma progression.^243^ Notably, nanomaterials offer significant advantages in drug delivery, especially in the context of intractable chronic conditions and cancers.^244^ By enhancing drug solubility, stability, and specificity, delivery systems such as lipid nanoparticles and ROS-responsive materials can effectively regulate LLPS-associated proteins by altering the local microenvironment.^245^ Moreover, magnetically guided nanoparticles enable targeted drug release, offering potential to reverse fibrosis or inflammation driven by aberrant LLPS.^246^ In the future, integrating LLPS-targeted strategies with nanomedicine could markedly improve the precision and efficacy of therapies for chronic liver diseases. To realize these prospects, a systematic dissection of LLPS-mediated molecular networks using multi-omics approaches and high-resolution imaging technologies will be crucial. Such efforts will not only deepen our mechanistic understanding but also facilitate the development of innovative tools and therapeutic modalities. Ultimately, this may lead to significant breakthroughs in the treatment of chronic liver diseases, improving patient outcomes and quality of life.

## Acknowledgments

The work was supported by the 10.13039/501100001809National Natural Science Foundation of China (82274185, 82374124, 82173874, and 82474164), the Leading Program for First Class Disciplines at Nanjing University of Traditional Chinese Medicine (ZYXYL2024-008), the National Key Laboratory of Traditional Chinese Medicine Pharmaceutical Process Control and Intelligent Manufacturing Technology Research Innovation Project (NZYSKL240102), the Traditional Chinese Medicine Cancer Poison Disclosure and Leading Project (AD202403), the 10.13039/501100004608Natural Science Foundation of Jiangsu Province (BK20230458), and the General Project of the Natural Science Research of Jiangsu Higher Education Institutions (23KJB310017).

## Author contributions

Writing – original draft and investigation, Q.X.; writing – original draft and investigation, T.H.; validation and investigation, G.Y.; validation and investigation, W.J.; validation and investigation, B.Z.; validation and investigation, Q.N.; writing – review and editing, W.F.; writing – review and editing, Z.Z.; writing – review and editing, Z.F.; writing – review and editing and supervision, Z.S.; writing – review and editing, supervision, and conceptualization, S.J.

## Declaration of interests

The authors declare no conflict of interest.
